# Development of a Health-Protective Drinking Water Level for Perchlorate

**DOI:** 10.1289/ehp.8684

**Published:** 2006-01-26

**Authors:** David Ting, Robert A. Howd, Anna M. Fan, George V. Alexeeff

**Affiliations:** Office of Environmental Health Hazard Assessment, California Environmental Protection Agency, Oakland, California, USA

**Keywords:** drinking water level, human health, iodine intake, NIS, perchlorate, risk assessment, sensitive subpopulation, thyroid hormone

## Abstract

We evaluated animal and human toxicity data for perchlorate and identified reduction of thyroidal iodide uptake as the critical end point in the development of a health-protective drinking water level [also known as the public health goal (PHG)] for the chemical. This work was performed under the drinking water program of the Office of Environmental Health Hazard Assessment of the California Environmental Protection Agency. For dose–response characterization, we applied benchmark-dose modeling to human data and determined a point of departure (the 95% lower confidence limit for 5% inhibition of iodide uptake) of 0.0037 mg/kg/day. A PHG of 6 ppb was calculated by using an uncertainty factor of 10, a relative source contribution of 60%, and exposure assumptions specific to pregnant women. The California Department of Health Services will use the PHG, together with other considerations such as economic impact and engineering feasibility, to develop a California maximum contaminant level for perchlorate. We consider the PHG to be adequately protective of sensitive subpopulations, including pregnant women, their fetuses, infants, and people with hypothyroidism.

Perchlorate is used mainly in the manufacture of solid propellants for rockets and missiles; it is also used in fireworks, road flares, blasting agents, and automobile air bags. Perchlorate is highly water soluble and stable at ambient temperature and pressure; it generally does not adsorb to organic or inorganic materials. Because of past disposal practices, extensive contamination of surface water and groundwater by perchlorate has been reported in California as well as other states. Recently, the U.S. Food and Drug Administration (U.S. FDA 2004) reported the detection of perchlorate in lettuce and dairy milk samples. Detections of perchlorate in lettuce, wheat, tomato, cucumber, cantaloupe, dairy milk, and human breast milk have also been reported by others (Environmental Working Group 2002; Jackson et al. 2005; Kirk et al. 2003, 2005; Téllez et al. 2005).

Because perchlorate was not recognized as an important environmental contaminant until 1997, there are no established federal or state regulatory standards. The California Office of Environmental Health Hazard Assessment (OEHHA) first released its draft risk assessment on perchlorate for public comment in March 2002. After extensive public and scientific peer reviews, OEHHA’s final risk assessment, “Public Health Goal for Perchlorate in Drinking Water,” was published in March 2004 (OEHHA 2004). In 2005 the National Academy of Sciences (NAS 2005) published its evaluation on the oral toxicity of perchlorate. The New Jersey Drinking Water Quality Institute (2005) and Massachusetts Department of Environmental Protection [Massachusetts DEP (2004b)] also released their risk assessments on the chemical. In this article we focus on the California efforts in developing a health-protective drinking water level for perchlorate, plus comments and discussion of more recent developments, which provide a wider perspective.

## Absorption and Toxicokinetics of Perchlorate

Data from human and animal studies indicate that perchlorate is readily absorbed from the gastrointestinal tract and distributed systemically with total body water. A higher concentration of perchlorate is associated with the thyroid than with other tissues. Perchlorate is essentially unmetabolized *in vivo* (Wolff 1998). When four patients were orally administered 200 mg of radiolabeled perchlorate (5 μCi), double labeled with ^36^Cl and ^18^O, most perchlorate was excreted unchanged in the urine, with the two labels (^36^Cl and ^18^O) remaining associated in the same molecule (Anbar et al. 1959). Because the specific iodide transport pump is present in the mammary gland, perchlorate is likely to be secreted into the breast milk. According to a human study reported by Greer et al. (2002) and an occupational study reported by Lamm et al. (1999), the biological half-life of perchlorate is approximately 8 hr.

## Mode of Action

One of the most important biological effects of perchlorate exposure at levels likely to be encountered in the environment is the reduction of iodide uptake by the thyroid. Iodide is actively transported via a transmembrane protein known as the sodium–iodide symporter (NIS) into thyroid cells. Iodide is a key component of thyroxine (T_4_) and triiodothyronine (T_3_), hormones that are used to regulate cell respiration, energy production, growth, and maturation of body tissues. Perchlorate competes with iodide for the NIS and at sufficiently high concentrations it can reduce or even completely block the uptake of iodide into thyroid cells. If there is a sustained decrease of iodine supply to the thyroid, synthesis and secretion of thyroid hormones can be impaired.

Levels of T_4_ and T_3_ in the blood are regulated by a negative feedback mechanism that helps the body to maintain hormone homeostasis. When thyroid hormone levels in the circulation are low, the hypothalamus stimulates the pituitary through thyrotropin-releasing hormone to produce thyroid-stimulating hormone (TSH), which in turn prompts the thyroid to produce more thyroid hormones. Prolonged stimulation of the thyroid can lead to hypertrophy and hyperplasia of thyroid cells, which can cause thyroid enlargement or goiter.

Iodine deficiency in pregnant women is a health concern, as it can lead to impaired growth and development in fetuses. The most severe form of congenital hypothyroidism is cretinism; less severe iodine deficiency can reduce maternal serum thyroid hormone levels and may subsequently impair fetal brain development (Bleichrodt and Born 1994; Glorieux et al. 1988; Haddow et al. 1999; Pop et al. 1999, 2003; Rovet et al. 1987; Tillotson et al. 1994; Vermiglio et al. 1990).

## Animal Toxicity Data

In acute and subchronic animal studies, perchlorate administered through the oral route (0.01–30 mg/kg/day) reduced uptake of iodide into the thyroid, perturbed thyroid hormone regulation, induced hypertrophy and hyperplasia in thyroid cells, and caused an increase in thyroid weight (Argus Research Laboratories 1998, 2001; Caldwell et al. 1995; Springborn Laboratories 1998; Yu et al. 2000).

In developmental and reproductive studies in rats, perchlorate at doses up to 30 mg/kg/ day did not affect fertility and pregnancy outcome measures. There were changes in thyroid weight, thyroid histopathology, and thyroid hormone levels in the dams or the offspring. Some changes in the fetal brain development were noted, but because of methodologic limitations, the interpretation of the data is unclear (Argus Research Laboratories 1998, 1999, 2001; Toxicology Excellence for Risk Assessment, unpublished data, 2001). In a two-generation rat study, two male pups in the 30-mg/kg/day dose group were found to have thyroid follicular-cell adenomas (Argus Research Laboratories 1999). Although the result was not statistically significant, it is noteworthy because of the low historical background incidence rate of the tumor and the relative short duration of exposure.

Ammonium perchlorate has been tested in a battery of *in vitro* and *in vivo* genotoxicity tests; perchlorate does not appear to be mutagenic or clastogenic (OEHHA 2004). A number of animal studies have shown that perchlorate at high doses (> 1,300 mg/kg/day) causes thyroid tumors in rodents (Gauss 1972; Kessler and Kruskemper 1966; Pajer and Kalisnik 1991). Because the occurrence of the tumors was preceded mostly by signs of thyroid hormone disruption and thyroid enlargement, these tumors are generally interpreted as being secondary to the anti-thyroid effects of perchlorate [OEHHA 2004; U.S. Environmental Protection Agency (U.S. EPA) 2002].

## Human Toxicity Data

In the 1960s, potassium perchlorate was used to treat patients with Graves disease. The therapeutic dose ranged from 500 to 2,000 mg/day; most of the treatments lasted several weeks, but in a few cases treatment as long as a year was reported (Crooks and Wayne 1960; Godley and Stanbury 1954; Morgans and Trotter 1960). In some patients receiving high doses, side effects such as skin rashes, nausea, gastrointestinal problems, and a serious blood disorder were noted (Barzilai and Sheinfeld 1966; Fawcett and Clarke 1961; Hobson 1961; Johnson and Moore 1961). Crooks and Wayne (1960) administered potassium perchlorate at 600–1,000 mg/day to a group of pregnant women who were suffering from hyperthyroidism and observed a slightly enlarged thyroid in 1 of the 12 infants born to the mothers. The enlarged thyroid returned to normal size in 6 weeks, and no other abnormalities were observed.

Many occupational (Braverman et al. 2005; Lamm et al. 1999) and ecologic studies (Brechner et al. 2000; Crump et al. 2000; Kelsh et al. 2003; Lamm and Doemland 1999; Li et al. 2000a, 2000b, 2001; Morgan and Cassady 2002; Schwartz 2001) investigated the effects of perchlorate exposure on thyroid function. Although most studies were negative, two studies showed a positive association (Brechner et al. 2000; Schwartz 2001). Confidence in the positive results is not high because of the small sample sizes, limited exposure data, and other methodologic issues.

The negative results of other ecologic studies that investigated the association between perchlorate in drinking water and abnormal thyroid functions in adults and neonates have also been questioned. Since the publication of these studies, perchlorate has been detected in lettuce and cow’s milk samples collected from various states (U.S. FDA 2004). It shows that perchlorate contamination is more widespread than previously thought and that it is not limited to drinking water. Thus, the “unexposed subjects” in the ecologic studies might also have been exposed to perchlorate. The potential misclassification of perchlorate exposure reduces the statistical power of, as well as the confidence in, the study results.

Many human studies have been conducted. Stanbury and Wyngaarden (1952) found that a single oral perchlorate dose as low as 2.2 mg caused detectable release of iodide from the thyroid and reported a positive correlation between perchlorate dose and the fraction of stored iodide discharged from the thyroid. At an oral dose of 900 mg/day for 4 or more weeks, Brabant et al. (1992) found that perchlorate caused a reduction of iodide stored in the thyroid and thyroid enlargement (U.S. EPA 2002), although there were no increases in TSH levels.

Lawrence et al. (2000, 2001) and Greer et al. (2002) administered perchlorate in drinking water to adult volunteers for 14 days and found a dose-related decrease in thyroidal iodide uptake. No change in serum T_4_, T_3_, or TSH levels was noted in all three studies. Because the Greer et al. study was selected for dose–response evaluation in our assessment, it is described in greater detail. Groups of euthyroid male and female subjects were exposed to perchlorate in water at 0.007, 0.02, 0.1, or 0.5 mg/kg for 14 days. The subjects were asked to drink one-quarter of the perchlorate dose at four time points spaced throughout each day. Thyroid iodide uptake was measured before (baseline), during, and after the 14-day exposure period. They found a statistically significant decrease in iodide uptake in all except the lowest-dose group. There was no sex difference. Uptakes measured on postexposure day 15 were not significantly different from the baseline, indicating the inhibitory effect of perchlorate is reversible.

## Hazard Identification

Based on the toxicity information reviewed, the most sensitive effect of perchlorate in humans is the reduction of thyroidal iodide uptake (Greer et al. 2002; Lawrence et al. 2000, 2001). Depending on the severity and duration of iodide uptake reduction, perchlorate can reduce the amount of iodide stored in the thyroid (Brabant et al. 1992) and cause thyroid enlargement (U.S. EPA 2002) in humans. At sufficiently high doses, perchlorate can cause histopathologic changes in the thyroid and induce thyroid tumors in rodents.

Although perchlorate has been shown to induce thyroid tumors in rodents, it is not believed that it poses a significant cancer risk to humans. Perchlorate has not been shown to be genotoxic. There is evidence that humans may not be as sensitive quantitatively to thyroid cancer from thyroid–pituitary disruption as rodents (U.S. EPA 1998). Thyroid hormones in rodents are not bound to T_4_-binding globulin as in humans; they have a higher rate of destruction and thus have to be replenished at a higher rate. Rodent thyroid is chronically stimulated and is more sensitive to chemicals that disrupt thyroid hormone balance (Hill et al. 1998).

Although there are limited human data on the health consequences of chronic exposure to perchlorate, health information related to iodine deficiency indicates that pregnant women and their fetuses are likely to be the most sensitive to the anti-thyroid effects of perchlorate. Glinoer (2001) suggested that pregnancy itself represents a stress on the thyroid hormonal system, and iodine deficiency can compound the problem. Results of a prospective study reported by Kung et al. (2000) showed that in a borderline iodine-sufficient area (median urinary iodine level = 9.8 μg/dL), pregnancy resulted in higher rates of maternal goitrogenesis as well as neonatal hypothyroxinemia and hyperthyrotrophinemia. It is important to note that thyroid enlargement in these women persisted and failed to revert completely even 3 months after delivery.

Several epidemiologic studies indicate that iodine deficiency during pregnancy may affect brain development and cause neurointellectual deficits in the offspring. The severity of effects depends on the timing as well as the severity of iodine deficiency and thyroid disorder (Morreale de Escobar et al. 2000). Evidence suggests that normal fetal brain development requires an adequate supply of maternal thyroid hormone throughout the first trimester, before the fetal thyroid begins to function (Hollowell and Hannon 1997). Even borderline maternal iodine deficiency, as observed in some European countries, may be accompanied by impaired school achievement in apparently normal children (Glinoer 2001).

## Dose–Response Evaluation

As discussed previously, the anti-thyroid effects of perchlorate appear to be similar in rodents and in humans. Quantitatively, however, rodents appear to be more sensitive. Several 14-day drinking water studies showed significant depression in serum T_3_ and T_4_ levels and elevation in serum TSH levels in rodents exposed to perchlorate doses as low as 0.01 or 0.1 mg/kg-day (Caldwell et al. 1995; Keil et al. 1998; Springborn Laboratories 1998; Yu et al. 2000). Similar human studies of the same exposure duration showed no changes in serum T_3_, T_4_, and TSH levels in volunteers exposed to doses up to 0.5 mg/kg/day (Greer et al. 2002; Lawrence et al. 2000). After reviewing the available toxicity studies, we considered the thyroidal iodide uptake data reported by Greer et al. (2002) to be the most appropriate for quantitative dose–response evaluation. The strengths of the study include both male and female human subjects, four appropriately spaced dose groups, a minimum of 7 subjects per dose group with a total of 37 subjects, a tightly controlled exposure regime, and thyroid function of the subjects measured before and after the exposure. Limitations of the study are short exposure duration and lack of report of iodine status of the subjects during the exposure.

Reduction of thyroidal iodide uptake was identified as the critical end point instead of changes in serum thyroid hormone levels or serum TSH levels because *a*) iodide uptake inhibition is a more clearly measurable phenomenon and *b*) the 14-day exposure in the Greer et al. (2002) study is an insufficient time to deplete thyroid iodine stores. Iodide-sufficient adults have enough iodide stored in the thyroid to support normal thyroid function for a few months. It is therefore not surprising that even at the highest dose of 0.5 mg/kg/day with > 65% reduction in iodide uptake, Greer et al. did not observe any changes in serum T_3_, T_4_, or TSH levels. Even at higher exposure levels, a correlation between perchlorate exposure and changes in serum T_3_, T_4_, or TSH levels may not be demonstrable. In human studies, an oral dose of 900 mg/day for 4 weeks had no effect on serum T_3_, T_4_, or TSH levels despite other adverse thyroid effects that were observed (Brabant et al. 1992; U.S. EPA 2002).

Use of serum T_3_, T_4_, or TSH level as the critical end point is also complicated by the likely large interindividual variation in the dose–response relationship. The perchlorate exposure thresholds for changes of these hormones are likely to depend on dietary iodide intake level, amount of iodide stored in the thyroid, preexisting stress on the thyroid, exposure to other goitrogens, and age.

The decision to choose a perchlorate level that does not reduce thyroidal iodide uptake for risk assessment is considered health protective. Iodide uptake reduction is the first step in a chain of events that if severe and prolonged can lead to changes in thyroid and pituitary hormone levels, histopathologic changes of the thyroid, and other adverse developmental effects. It is believed that if perchlorate exposure is kept at a level that does not affect iodide uptake, then all the subsequent adverse health effects can be prevented.

Another advantage of choosing inhibition of thyroidal iodide uptake as the end point is that it can minimize the effect of perchlorate on NIS in nonthyroidal tissues. Besides the thyroid, NIS has been found in stomach, lactating mammary gland, and placenta and to a lower extent in small intestine, skin, and brain (Perron et al. 2001). Because breast milk is the sole source of nutrient for many infants, the potential of perchlorate to reduce the secretion of iodide into the breast milk needs to be considered.

**Figure 1:**
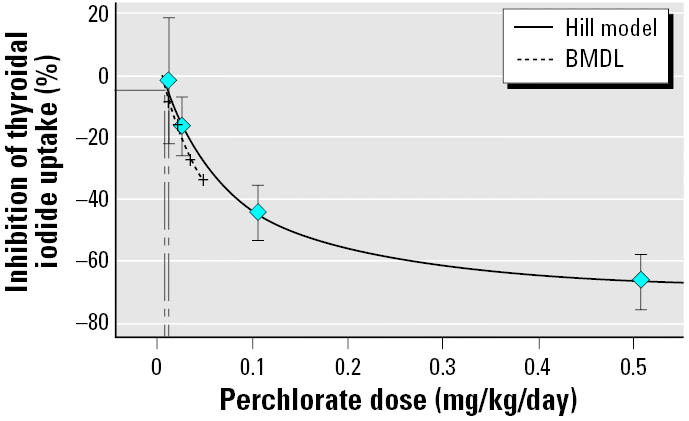
Analysis of the Greer et al. (2002) data by the BMD approach. Error bars represent the 95% CI of the estimated mean.

We employed the Benchmark Dose Software (version 1.3.1; U.S. EPA 2000) to perform the dose–response evaluation on the data (Table 1) reported by Greer et al. (2002). A benchmark dose (BMD) approach was used because it uses all the data in the study, it is less affected by the spacing of the doses, and it accounts for the variability of the data as well as the slope of the dose–response relationship. We found that the Hill model adequately describes the Greer et al. data (goodness of fit test, *p* = 0.46), shown plotted in Figure 1. The model was run with intercept set to zero, power parameter restricted to be greater than one, and the assumption of a constant variance. The fit is generally considered adequate when the *p*-value is > 0.05. The form of the response function estimated by the model is as follows:


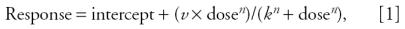


where intercept = 0, *v* = −73.4469, *n* = 1.15067, and *k* = 0.0663651.

In choosing an appropriate benchmark response level (BMR) for BMD modeling, we consulted the U.S. EPA (2000) guideline and determined that none of its suggestions are applicable in this situation because it is not obvious what level of thyroidal iodide uptake reduction should be considered biologically significant or adverse, and there is no control group in the Greer et al. (2002) study. Instead, we experimented by setting the BMR to a reduction of *a*) the mean standard deviation (14%) of all the dosed groups in the study, *b*) 10%, or *c*) 5%, and found that the BMD corresponds to a calculated dose of 0.0188 mg/kg/day, 0.013 mg/kg/day, or 0.0068 mg/kg/day, respectively. Because the calculated dose of 0.0188 mg/kg/day is close to the second lowest dose (0.02 mg/kg/day) of the study (Table 1), it can be considered to give a positive response. The calculated dose of 0.0068 mg/kg/day is close to the lowest dose (0.007 mg/kg/day) of the study (Table 1); it can be considered to give a negative response. Without additional data and not knowing the standard deviation of the control group, it is difficult to determine if the calculated dose of 0.013 mg/kg/day (BMR = −10%) is a lowest observed effect level (LOEL) or a no observed effect level (NOEL). Given the options, we decided to use −5% as the BMR and considered it a NOEL. Using the parameters described, we found that the BMD corresponds to 0.0068 mg/kg/day and that the lower limit of a one-sided 95% confidence interval on the BMD or the lower confidence limit of the BMD (BMDL) corresponds to 0.0037 mg/kg/day. In the risk assessment, we used the BMDL as the point of departure because it takes into consideration the limited sample size of the study and the variability exhibited in the data.

## Development of a Health-Protective Drinking Water Level

Because pregnant women and their fetuses are identified as the sensitive subgroups, we used the following equation to estimate a health-protective concentration for drinking water (*C*, in milligrams per liter):


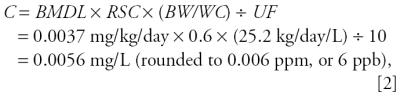


where *BMDL* is the lower limit of a one-sided 95% confidence interval of a perchlorate dose that reduces mean thyroidal iodide uptake by 5%; *RSC* is the relative source contribution (a value of 60% is used for pregnant women because of the detection of perchlorate in farm produce and cow’s milk); (*BW/WC*) is the ratio of body weight (kilograms) and tap water consumption rate (liters per day); the ratio for the 95th percentile of the pregnant woman population is estimated to be 25.2 kg/day/L; OEHHA 2000); and *UF* is an uncertainty factor of 10 to account for interindividual variability.

The water concentration resulting from this calculation, 6 ppb, is judged adequate to protect all individuals, including potential sensitive subpopulations, from adverse health effects of perchlorate, from short-term to chronic exposures. An uncertainty factor of 10 for interindividual variability is used because the subject population in the Greer et al. (2002) study is small (37 subjects) and did not include pregnant women, infants, or individuals with thyroid problems. Dietary iodine intake and thyroidal iodide uptake are known to vary among individuals; they are affected by the type of food one eats (some food is rich in iodine, whereas other foods contain goitrogens), smoking habits (tobacco smoke contains goitrogens), medication (e.g., lithium), and exposure to environmental contaminants (e.g., nitrate, poly-chlorinated biphenyls, and dioxins). The interindividual variability in the general population is likely to be greater than that shown by the study population.

Preliminary survey results indicate that perchlorate is detected in lettuce, wheat, tomato, cantaloupe, cucumber, and cow’s milk (Jackson et al. 2005; Kirk et al. 2003; Sharp 2004; Smith and Jackson 2003; U.S. FDA 2004). Perchlorate has also been detected in human breast milk samples (Kirk et al. 2005; Téllez et al. 2005), thus confirming the viability of the breast milk exposure pathway as well as indicating significant neonatal exposures to perchlorate from sources other than drinking water. However, the data on food sources were inadequate for a precise calculation of the contribution of perchlorate from drinking water versus food for pregnant women, the presumed most sensitive population (with their fetuses). On the basis of the available data, we assumed that most of the perchlorate exposure would come from drinking water and determined that the most appropriate relative source contribution is 60%.

Valentin-Blasini et al. (2005) analyzed urine samples collected from 61 adults in an area with no known perchlorate contamination (Atlanta, Georgia) and estimated that the perchlorate in urine ranged from 0.66 to 21 ng/mL with a median level of 3.2 ng/mL (7.8 μg perchlorate/g of creatinine). Because the researchers found no perchlorate in the area tap water, one can assume the perchlorate in the urine was mainly derived from food or sources other than drinking water. Based on the public health goal (PHG) value of 6 ppb (micrograms per liter) and a water exposure measure of 25.2 kg/day/L (Equation 2), a perchlorate dose of 0.24 μg/kg/day from drinking water exposure can be calculated. When this dose estimate is combined with the median estimate of 0.16 μg/kg/day provided by an author of that study (Blount B, personal communication), one obtains an RSC of 60% for perchlorate from water. This rough calculation supports the use of 60% as a reasonable estimate of the RSC used in our risk assessment (although the results of this study were made available to us after our initial calculations). An RSC of 20% from water (80% from other sources, including food), the default recommended by the U.S. EPA, would be more applicable to average exposures, which are less than 6 ppb, but was not representative of our thinking in this case.

It has been suggested that an additional uncertainty factor of 3 is needed to account for the short exposure duration of the Greer et al. (2002) study. However, there is evidence that iodine uptake is inhibited very quickly after exposure begins and that the inhibition does not worsen as exposure continues. In three of the dose groups, 0.02 mg/kg/day, 0.1 mg/kg/day, and 0.5 mg/kg/day, the degree of inhibition on day 2 was similar to inhibition of day 14 (Greer et al. 2002). Furthermore, it can be argued that if there is no reduction in acute thyroidal iodide uptake, there will be no reduction in stored iodide, and extending the exposure duration is not likely to affect thyroid function. For this reason, we concluded that no additional factor is necessary to account for the short exposure duration of the study.

## Discussion and Conclusion

In our human health risk assessment of perchlorate, we identified the reduction of thyroidal iodide uptake as the critical end point. It is reasoned that if this undesirable effect can be avoided, then all the subsequent health effects related to thyroid hormone disruption can be prevented. We applied BMD modeling techniques to a set of human data reported by Greer et al. (2002) and determined a BMDL (equivalent to a NOEL) of 0.0037 mg/kg/day. Dividing by an uncertainty factor of 10 for human variability, a health-protective daily dose of 0.00037 mg/kg/day would be derived from this exercise. A PHG of 6 ppb was calculated by using the health-protective daily dose, a relative source contribution of 60%, and exposure assumptions specific to pregnant women.

Because the selected end point is related to a physical property of a membrane protein, the NIS, the interindividual variability is likely to be less than that of changes in serum thyroid hormone or TSH level. The threshold of the chosen end point is also less likely to be affected by exposure duration, iodide status, and the physiologic condition of the subject. Given these considerations and the fact that the critical end point was based on an early effect in the chain of possible perchlorate effects, derived from human studies, we concluded that the uncertainty factor of 10 would be adequate.

The recent analysis of perchlorate data by a committee of the National Academy of Sciences (NAS 2005) also used the data of Greer et al. (2002) as a basis for its risk assessment. The NAS committee assumed that the lowest perchlorate dose (0.007 mg/kg/day) in the Greer et al. study represented a NOEL for iodide uptake inhibition and estimated a reference dose (RfD) by dividing this NOEL by an uncertainty factor of 10, corresponding to an equivalent dose of 0.0007 mg/kg/day. The committee acknowledged the utility of BMD modeling of the Greer et al. data but emphasized that there would be some difficulty choosing among the various approaches already published. Therefore it chose the NOEL approach for its transparency. The NAS committee did not proceed to estimate a health-protective level of perchlorate in drinking water from this RfD because this was not one of the charges in its review. However, if a health-protective level of perchlorate in drinking water were estimated from this RfD using the U.S. EPA default procedures, including a default RSC of 0.2, the following equation would result:


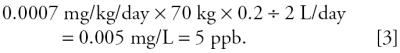


This result is essentially the same result as that derived by our risk assessment approach, although our use of the BMD method to determine a statistically more robust equivalent of NOEL seems scientifically preferable. It is not clear whether the U.S. EPA will use this approach if it proceeds to establish a maximum contaminant level goal for perchlorate in drinking water. However, it should be noted that this is the approach used by the State of New Jersey in their newly proposed perchlorate rule (New Jersey Drinking Water Quality Institute 2005).

A recent report by Ginsberg and Rice (2005) criticized the NAS recommendation, arguing that the lowest dose of the Greer et al. (2002) study does not represent a NOEL. They suggested that this dose should be treated as an LOEL because four of the seven subjects in the group showed a decrease in thyroidal iodide uptake. Ginsberg and Rice also suggested that perhaps the four subjects who appeared to show a response to perchlorate constitute a sensitive group because their thyroidal iodide uptakes were consistently higher than the rest, with or without perchlorate exposure. The Massachusetts Department of Environmental Protection interpreted these data in the same way in their recent perchlorate risk assessment (Massachusetts DEP 2004b). These factors, it is argued, justify a much lower health-protective standard. Looking at the Greer et al. data, we find it difficult to say whether there are two distinct subgroups or whether the difference is just a manifestation of normal interindividual variability. Because of the small number of subjects and the relatively large variability observed in the lowest-dose group, we decided not to use the NOEL/LOEL approach for dose–response characterization. Instead, we chose to use the BMD approach. Doing so addresses these issues and uses all the data in the study, not just those in the lowest-dose group.

Use of iodide uptake inhibition as the critical end point for perchlorate risk assessment has also been criticized for being too stringent. It has been argued that reduction of iodine uptake is not an adverse effect, but rather a precursor to an adverse effect (Strawson et al. 2004). It has been suggested that the highest dose in the Greer et al. (2002) study should be identified as the no observed adverse effect level (NOAEL) instead because after 14 days of exposure at 0.5 mg/kg/day, no significant changes in serum T_3_, T_4_, or TSH levels were observed in the exposed subjects. We disagree with this determination because even with a complete inhibition of thyroidal iodine uptake, the amount of iodide stored in an iodine-replete adult can sustain normal thyroid function for several months (Greer et al. 2002). It is therefore likely that if the perchlorate exposure at the 0.5 mg/kg/day level were prolonged, there could be an impact on the thyroid function. As pointed out previously, even under a controlled clinical situation and at high exposure levels, it is not easy to establish a relationship between serum T_3_, T_4_, or TSH levels and perchlorate exposure (Brabant et al. 1992). The thresholds for the hormonal changes may be different for different individuals because of variations in dietary iodide intake, exposure to other goitrogens, age, and physiologic conditions. Also, when an individual suffers from a mild iodide deficiency, the drop in serum T_3_ and T_4_ and the rise of serum TSH are only transitory. These hormonal changes make the thyroid function at a higher level. When a new equilibrium is reached, the serum T_3_, T_4_, and TSH levels return to normal. Indeed, the NAS committee stated that under such conditions, thyroid enlargement might be the only evidence that there had been a change of serum thyroid hormones and TSH at an earlier time (NAS 2005).

Strawson et al. (2004) argued that serum T_4_ decrease is the critical effect of perchlorate, which would be preferred for use in the risk assessment. However, they acknowledge that decreases in serum T_4_ have not been observed in available human studies and that therefore other approaches seem to be more useful. Modeling the iodide uptake data of Greer et al. (2002), Strawson et al. (2004) derived a dose of 0.006 mg/kg/day based on a 10% iodide uptake inhibition level. They compare this dose with a “free-standing” NOAEL (the highest exposure had no significant effect) of 0.006 mg/kg/day in a human epidemiology study of Chilean children (Crump et al. 2000). Their conclusion is that, taken together, these data define the potential effects in a sensitive population quite well and justify the use of a small uncertainty factor of 3. This results in an RfD of 0.002 mg/kg/day. Using the same exposure parameters as described in Equation 3, this would result in a health-protective level in drinking water of 14 ppb.

An opinion has been expressed that reduction of iodine uptake is not the best end point for risk assessment because the effect is mundane and can be caused by other chemicals (Belzer RB, Bruce GM, Peterson MK, Pleus RC, unpublished data, 2003; Bruce et al. 2005). Nitrate and thiocyanate also compete with iodide for the NIS, and they are commonly found in food and the environment. These chemicals are considerably less potent than perchlorate as inhibitors of iodide uptake (Lambers et al. 2000) but often occur at higher concentrations. It is argued that it is unfair to regulate perchlorate at a level that does not cause reduction in thyroidal iodide uptake but at the same time allow exposures to nitrate and thiocyanate at levels where such an effect may occur. However, the presence of other inhibitors in the environment increases the need for vigilance against additional environmental contaminants, and California law requires consideration of the effects of multiple chemicals that act similarly. Allocation of an iodine-inhibition potential to chemicals consumed in foods (which provide some benefits) might also be considered to take precedence over pollutants consumed in drinking water.

OEHHA’s PHG has also been viewed as not sufficiently health protective (Madsen and Jahagirdar 2005; Massachusetts DEP 2004a). It has been suggested that a larger uncertainty factor should be used to account for the small number of subjects in the lowest-dose group of the Greer et al. (2002) study, the lack of quality long-term exposure data, higher exposure of infants and small children on a bodyweight basis, and special susceptibility of infants’ brains to thyroid hormone disruption. However, these opinions do not address the fact that iodide uptake inhibition is not in and of itself an adverse health effect. The daily dose of perchlorate required to have an eventual effect on thyroid hormone production is not well established but is certainly much higher than the health-protective dose estimated here, by the NAS (2005), or by Strawson et al. (2004), largely on the basis of prevention of iodide uptake inhibition. The BMD modeling of the human iodide uptake inhibition data of Greer et al. (2002) provides an adequately health-protective end point. An additional 10-fold uncertainty factor addresses the limitations related to this study as well as the existence of other goitrogens in the environment. Based on our current understanding of the toxicokinetics of perchlorate (Clewell et al. 2003; U.S. EPA 2002), the internal perchlorate doses (expressed as area under the curve of blood concentration) are similar in infants and adults, at a given water concentration. This is because perchlorate is not metabolized or retained by the body to any significant extent, and the higher intake rate (on a body-weight basis) of infants is likely to be balanced by a higher excretion rate (on a body-weight basis).

The approach described here makes the best use of the available toxicologic data and uses a reasonable approach in choosing a health-protective level for perchlorate in drinking water. By choosing a precursor effect to define the point of departure in the risk assessment, the risk assessment is likely to err on the side of caution. This is partially offset by the choice of a relatively small uncertainty factor. Thus, we do not feel that the resulting recommended health-protective level of 6 ppb perchlorate in drinking water is overprotective, given the size of uncertainty factor chosen and the various remaining uncertainties in the data. As new information becomes available, the health-protective level will be subject to revision under California law.

## Figures and Tables

**Table 1:** Reduction of thyroid radioiodine uptake after a 14-day exposure to perchlorate.

Average dose (mg/kg/day)	Change in radioactive iodine uptake by the thyroid (%)after 24 hr of dosing (mean ± SD)	No. of subjects in each dose group
0.007	−1.8 ± 22.0	7
0.02	−16.4 ± 12.8*	10
0.1	−44.7 ± 12.3*	10
0.5	−67.1 ± 12.1*	10

Data from Greer et al. (2002) and Goodman G (personal communication).

* Statistically significant, *p* < 0.005 (pairwise comparison with baseline).
